# Comment on: Non-Cholesterol Sterol Concentrations as Biomarkers for Cholesterol Absorption and Synthesis in Different Metabolic Disorders: A Systematic Review

**DOI:** 10.3390/nu11040780

**Published:** 2019-04-04

**Authors:** Rime Jebai, Semiu O. Gbadamosi, Liliana Nassar Gorra, Purnima Madhivanan

**Affiliations:** Department of Epidemiology, Robert Stempel College of Public Health and Social Work, Florida International University, 11200 SW 8th Street, Miami, FL 33199, USA; sgbadamo@fiu.edu (S.O.G.); lnass005@fiu.edu (L.N.G.); Pmadhiva@fiu.edu (P.M.)

The use of non-cholesterol sterols as biomarkers for cholesterol metabolism is well established in health-related topics. However, their roles in various metabolic conditions need to be investigated, especially since dietary interventions and pharmacological treatments can affect cholesterol metabolism due to their underlying mechanisms. This rationale highlights the importance of the systematic review entitled “Non-Cholesterol Sterol Concentrations as Biomarkers for Cholesterol Absorption and Synthesis in Different Metabolic Disorders: A Systematic Review” by Mashnafi et al. published in a recent edition of Nutrients [1]. Nevertheless, several methodological flaws exist which may have an impact on the quality of the review and affect the utility of the findings by clinicians and their applicability in dietary interventions.

Mashnafi et al. retrieved the articles from three important databases; however, their search was limited to only English language and cross-sectional study designs. These limitations narrow the search literature and risk missing many relevant studies. Various other languages and study designs could have improved the comprehensiveness of the findings [2]. In addition, they did not report their search strategy, nor did they state the period for article selection, thus limiting the reproducibility of their findings for further research. The replicability of a comprehensive search strategy ensures methodological consistency and improves the quality of scientific evidence [3]. 

Moreover, there is no evidence to demonstrate that the authors registered their protocol or proof to show that they used any structured guidelines in the analysis such as the Preferred Reporting Items for Systematic Reviews and Meta-Analyses guidelines, which could ensure adequate reporting of results. Following these guidelines could minimize bias, improve the quality of the review, and enable future updates [4,5]. While it is important to evaluate the quality of original studies [5], the authors failed to assess the risk of confounding, selection, or attrition bias, which otherwise may have improved the credibility and value of the systematic review.

Well-designed reviews must incorporate relevant articles and use transparent, reproducible, and objective methodological approaches in order to be useful for informing clinical decisions and guiding policymaking. However, the presence of methodological flaws affects the quality of the evidence and the accuracy of conclusions. Therefore, to obtain a high-quality systematic review, authors should follow guidelines such as those recommended by the Cochrane Collaborations or the International Committee of Medical Journal Editors.
